# Clinical, trichoscopic, and histological characteristics of 46 hispanic men with fibrosing alopecia in a pattern distribution: a retrospective multicenter analysis

**DOI:** 10.3389/fmed.2026.1746147

**Published:** 2026-02-26

**Authors:** Luis E. Sánchez-Dueñas, Irene M. Rodriguez-Escamilla, Joel A. Ramírez-Sánchez, Daniel Jimenez-Zaragoza, Guillermo Solis-Ledesma, Guillermo A. Guerrero-González, Miguel Marti-Machado, Mariana Lavia, Sonia S. Ocampo-Garza, Lizet K. Rojano-Fritz, Aldo Gálvez-Canseco, Leslie E. Rocha-Mendez, Nicole Orendain-Koch

**Affiliations:** 1 Department of Dermatology, Dermatologic Institute of Jalisco “Dr. José Barba Rubio”, University of Guadalajara, Zapopan, Mexico; 2 Department of Dermatology, General Hospital “Dr. Manuel Gea Gonzalez”, Mexico City, Mexico; 3 Department of Dermatopathology, Dermatologic Institute of Jalisco “Dr. José Barba Rubio”, University of Guadalajara, Zapopan, Mexico; 4 Private Practitioner, Monterrey, Mexico; 5 TRICOMED, Buenos Aires, Argentina; 6 Department of Dermatology, Autonomous University of Nuevo, University Hospital “Dr. José Eleuterio González”, Monterrey, Mexico; 7 Private Practitioner, Barranquilla, Colombia; 8 Private Practitioner, Lima, Peru; 9 Private Practitioner, León, Guanajuato, Mexico; 10 Dermika Laser Dermatologic Center, Guadalajara, Mexico

**Keywords:** cicatricial alopecia, fibrosing alopecia in a pattern distribution, hispanic population, multicenter study, trichoscopy

## Abstract

**Background:**

Fibrosing alopecia in a pattern distribution (FAPD) is an infrequent presentation of primary cicatricial alopecia with clinical, histopathological and trichoscopic features of Androgenetic Alopecia (AGA), as well as Lichen Planopilaris (LPP).

**Objectives:**

The aim of this study is to describe the clinical, trichoscopic and histopathological features of 46 hispanic male patients with FAPD.

**Methods:**

This was a retrospective study from 8 dermatological centers across four countries: Argentina, Colombia, Mexico, and Peru. Patients with clinical diagnosis of FAPD performed by 12 dermatologists experienced in trichology from 2015 to 2022 were included.

**Results:**

Forty-six male patients were identified, with a mean age of 39. The age of onset ranged from 20 to 63 years. 85% of the patients (*n* = 39) had a family history of AGA. In terms of clinical characteristics, the Male Pattern of Hair Loss (MPHL) was the most common (65%). All patients showed a symmetrical distribution of the alopecia. Regarding the trichoscopic characteristics, the most frequent finding was perifollicular desquamation (96%), while most common histological finding was concentric perifollicular lamellar fibrosis (100%). FAPD was the initial clinical suspicion in only 6 patients (13%), and androgenetic alopecia was the primary initial suspicion (56.5%).

**Conclusion:**

The age of onset of FAPD appears to be earlier in Latin American male patients compared with European and North American male patients. Most male patients with FAPD clinically resemble AGA in their classic pattern of hair loss.

## 1 Introduction

Fibrosing alopecia in a pattern distribution (FAPD) is an infrequent presentation of primary cicatricial alopecia with clinical, histopathological, and trichoscopic features of both Androgenetic Alopecia (AGA) and Lichen Planopilaris (LPP) (1, 2). It was first described by Zinkernagel and Trüeb in 2000, when they mentioned that FAPD is related to AGA in its pathogenesis and distribution, with a diffuse hair loss pattern that progresses to a cicatricial and lichenoid reaction (3). Their findings were supported by clinical response with an antiandrogen that decreased hair loss and reduced inflammation (3). This alopecia is more prevalent among women than men, but while males manifest it at an earlier age, females tend to manifest it in the postmenopausal period (1–3). Our objective was to describe the clinical, trichoscopic, and histopathological features of 46 Hispanic male patients with FAPD.

## 2 Method

### 2.1 Subjects

We included patients with clinical, trichoscopic, and histopathological diagnoses of FAPD who had a minimum of 6 months of medical follow-up conducted by a team of 12 dermatologists and trichologists with specialized expertise in the disease from 8 dermatological centers across four countries: Argentina, Colombia, Mexico, and Peru, spanning from 2015 to 2022. We included 46 patients. The diagnosis of FAPD was made by dermatologists experienced in trichology. All included patients were required to meet all three diagnostic domains: clinical, trichoscopic, and histopathological criteria. Specifically, diagnosis required: (i) Clinical criteria: a patterned distribution of scalp hair loss consistent with male or female pattern hair loss (MPHL or FPHL); (ii) Trichoscopic criteria: the presence of at least one characteristic feature, including perifollicular scaling, loss of follicular openings, hair shaft diameter variability, single-hair follicular units, or perifollicular erythema; and (iii) Histopathological criteria: evidence of concentric perifollicular lamellar fibrosis (1–3). Only patients with at least 6 months of medical follow-up were included. All cases were reviewed and diagnosed by dermatologists with expertise in trichology using the same predefined criteria across the eight participating centers, ensuring diagnostic consistency.

### 2.2 Data collection

We evaluated demographic characteristics: patients’ age, sex, disease duration, clinical manifestation: pattern of scalp hair loss MPHL or FPHL, trichoscopic findings: perifollicular scaling, loss of follicular openings, variability of hair shaft diameter and single hair follicles, perifollicular erythema, trichoscopic analysis was carried out using either manual dermoscopy devices (DermLite CA USA) or digital dermoscopy equipment (FotoFinder Dermoscope® Germany). Histopathological findings: concentric perifollicular lamellar fibrosis, lymphohistiocytic infiltrate, fibrotic follicular tracts and follicular miniaturization.

### 2.3 Statistical analyses

Descriptive statistics included measures of central tendency, and frequency, medians, ranges, and proportions.

## 3 Results

### 3.1 Patient characteristics

All forty-six male patients were included in the analysis. The mean age was 39 years (standard deviation 11.23 years). The age of onset ranged from 20 to 63. 85% of the patients (*n* = 39) had a family history of AGA.

Patients with Male Pattern Hair Loss (MPHL) accounted for 65.2% (*n* = 30) of our sample and were classified according to the Norwood–Hamilton (NH) scale: 22% were type III (*n* = 10), 22% type IV (*n* = 10), 6.5% type V (*n* = 3), and 15% type VI (*n* = 7).

**Figure 1:**
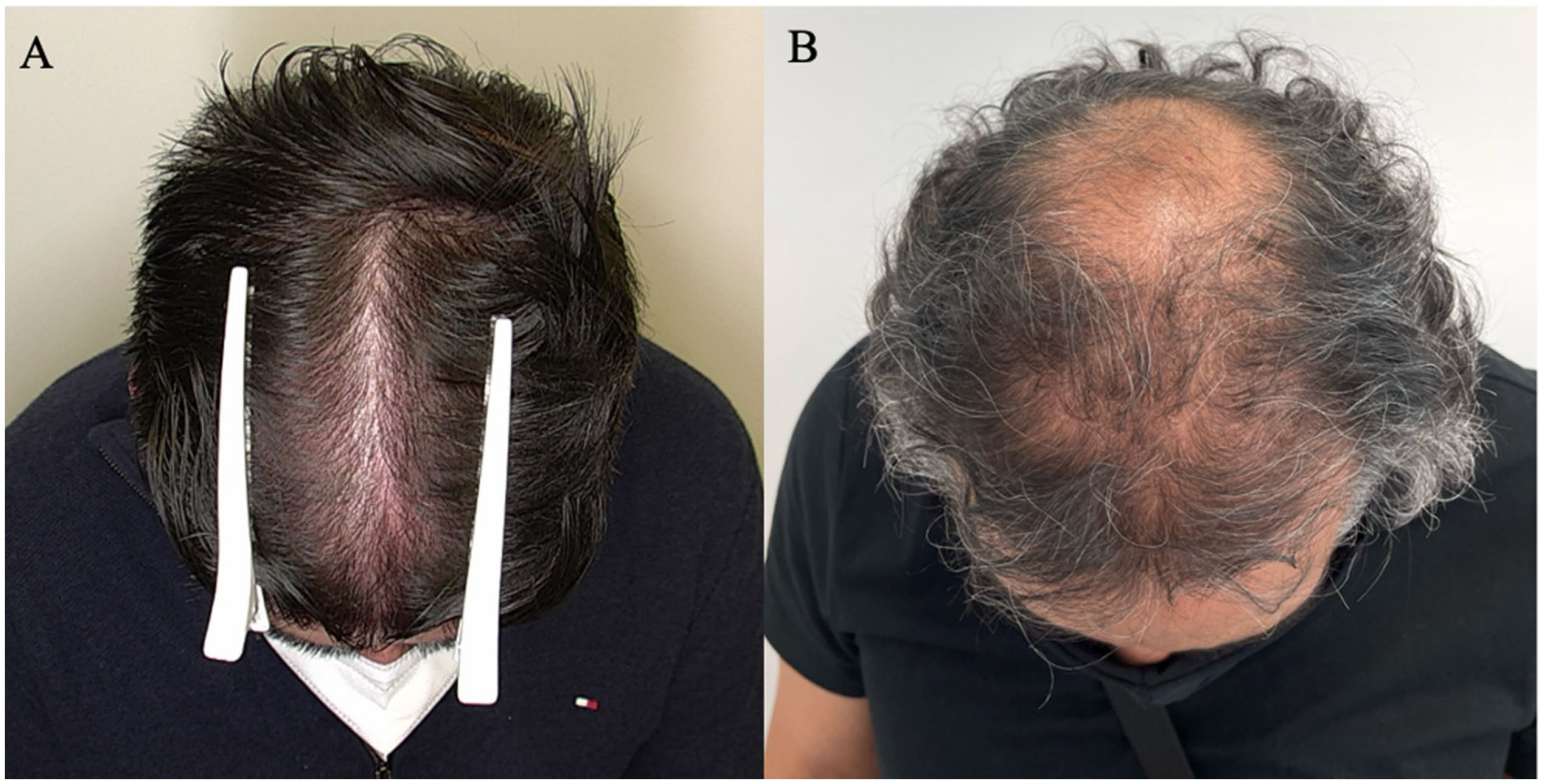
Example of clinical presentation of patterns: **(A)** FPHL, S grade III; **(B)** MPHL, HN type IV.

Patients with Female Pattern Hair Loss (FPHL) represented 34.8% (*n* = 16) of our sample and were classified according to the Sinclair (S) scale: 4.3% were grade II (*n* = 2), 13% grade III (*n* = 6), and 17% grade IV (*n* = 8). Both patterns are illustrated in Figure 1.

Regarding the symmetry of the alopecia, all patients had a symmetrical distribution, and only 4.3% (*n* = 2) had other body areas affected. Lichen planus in any body area occurred in three patients (6.5%).

FAPD was initially suspected in only 13% (*n* = 6) of the cases. The suspected initial diagnosis was AGA in 56.5% (*n* = 26), and 26% (*n* = 12) had a non-specific diagnosis. Additionally, seborrheic dermatitis (SD) was thought to be associated with AGA and FAPD in 3 patients. The main complaint at consultation was a decrease in hair density in 63% (*n* = 29) and a poor response to previous hair loss treatments in 72% (*n* = 33). Interestingly, pruritus and trichodynia were virtually absent in our sample, with only two patients (4%) reporting these symptoms.

### 3.2 Trichoscopic characteristics

Trichoscopy was performed in all patients. The most prevalent findings were perifollicular scaling in 96% (*n* = 44), followed by loss of follicular openings and variability of hair shaft diameter, both in 89% (*n* = 41), predominance of single hair follicles in 83% (*n* = 38), and perifollicular erythema in 70% (*n* = 32).

### 3.3 Histopathology characteristics

Upon analyzing the histopathology data, we found that only 24% of the biopsies (*n* = 11) had two samples taken for both vertical and horizontal sections, 67% favored only the vertical section (*n* = 31), and 8.7% only the horizontal section (*n* = 4). Concentric perifollicular lamellar fibrosis was present in all 46 cases, lymphohistiocytic infiltrate in 98% (*n* = 45), fibrotic follicular tracts in 66% (*n* = 29), and follicular miniaturization in 54% (*n* = 29).

In terms of treatment, 98% received dutasteride 0.5 mg (*n* = 45), 87% received oral minoxidil (*n* = 40), and 50% received topical steroids (*n* = 23). At 6 months, the response to treatment was: improvement in 32% of patients (*n* = 14), stabilization in 34% (*n* = 15), and worsening in 34% (*n* = 15). Two patients were lost to follow-up.

## 4 Discussion

FAPD is a rare form of primary cicatricial alopecia with a lichenoid inflammation pattern, sharing clinical, trichoscopic, and histological features of both AGA and LPP. This distinctive condition was initially described by Zinkernagel and Trüeb in 2000 (1–3).

Most case series exhibit a marked female predominance but an earlier age of onset among males, who are typically affected in their fifth decade. This was outlined in the original paper, where only 4 out of 19 patients were men, with a mean age of 54 years old (the mean age among women was 59 years). Another international multicenter study had similar findings: 11% were men (*n* = 57), with a mean age of 57 vs. 59 in women (4). A case series of 13 patients from Chile, however, described an even lower mean age of 37 years among the four men included (ranging from 28 to 46 years) (5). Similarly, a Japanese publication on FAPD affecting 7 males (from a sample of 24) found a mean age of 26.57 years (ranging from 17 to 40 years) (6). Such results correlate with ours and suggest that male patients in Latin America, perhaps similar to the Asian population, may have an earlier age of presentation than Europeans and North Americans (1, 3–6).

The pathogenesis of FAPD is not fully understood, with some experts considering it a variant of lichen planopilaris or a type of primary lymphocytic cicatricial alopecia (1, 2). The latter is characterized by a lichenoid reaction to the upper part of the hair follicle, triggering perifollicular inflammation and fibrous follicular tracts (1, 2). It is also theorized that in immunogenetically susceptible patients, hair follicles altered by the progression of AGA may produce a lichenoid reaction when exposed to an antigenic stimulus (1).

Contrary to what was published by Zinkernagel and Trüeb, who identified 13 cases of FPHL and only 6 of MPHL, MPHL predominated in our sample (3). This is important since FPHL affecting the centroparietal area is considered the most common presentation (1–3). In their paper, 75% of the men with MPHL had a Hamilton—Norwood scale stages III and IV (3). It also indicates that, despite being less common in males than females, FAPD resembles AGA with progressive bitemporal, frontal, and vertex recession as described in other publications (1–3).

**Figure 2:**
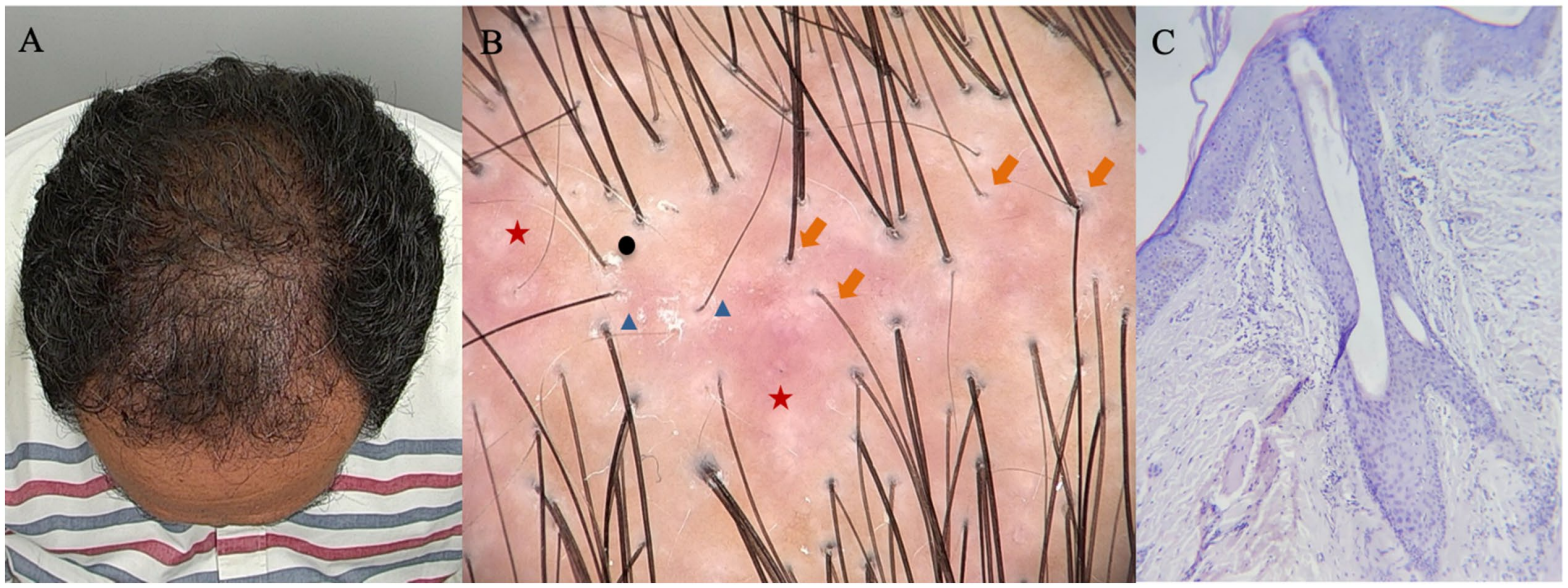
45-year-old male with FAPD: **(A)** HN type VI; **(B)** Trichoscopy with perifollicular scale (black circle), loss of follicular openings (red star), variability of hair shaft diameter (orange arrow), and single hair follicles (blue triangle); **(C)** Biopsy with presence of lymphohistiocytic infiltrate predominantly in the upper region of the.

**Figure 3:**
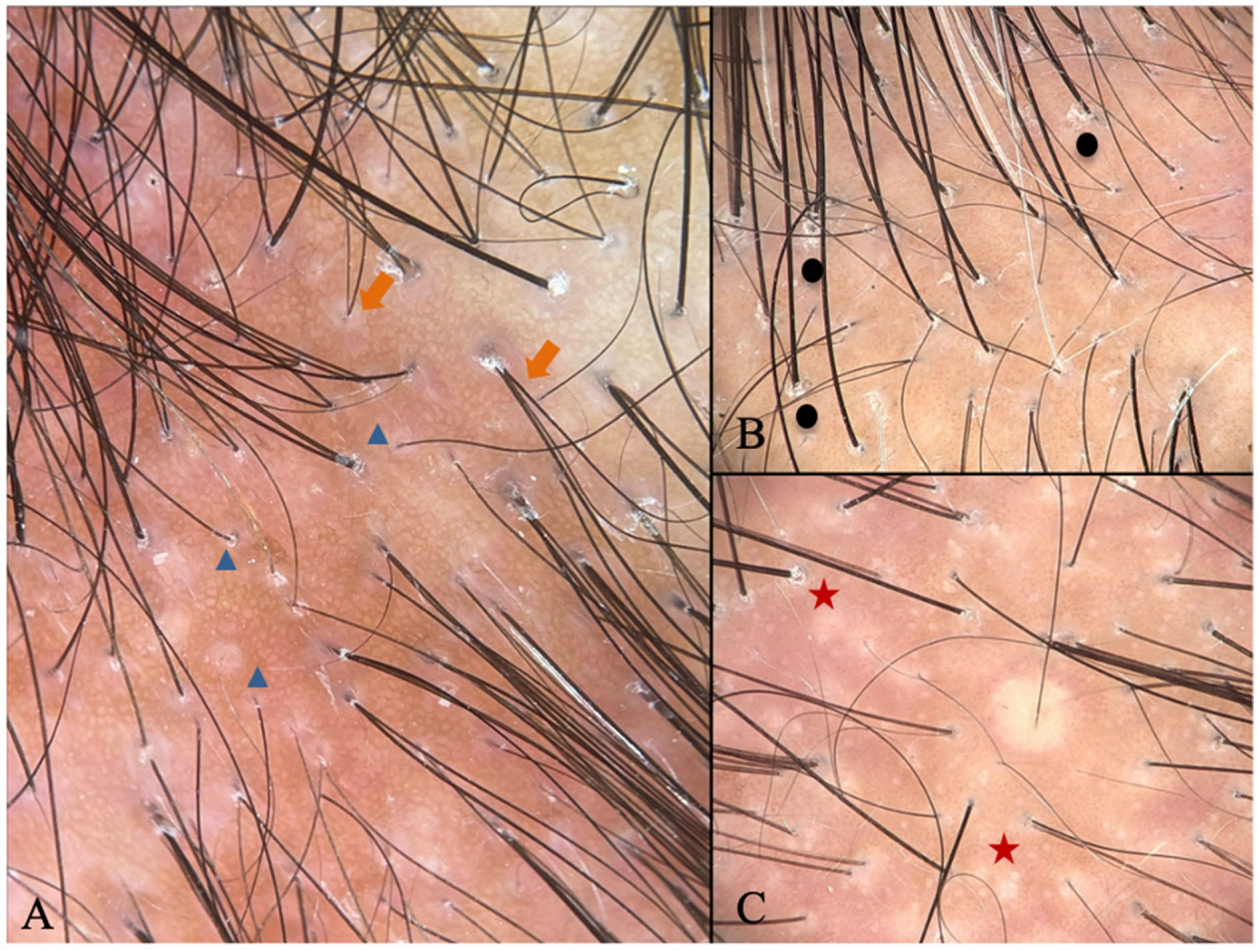
Trichoscopy characteristic of FAPD: **(A)** Variability of hair shaft diameter (orange arrow) and single hair follicles (blue triangle); **(B)** perifollicular scale (black circle); **(C)** loss of follicular openings (red star).

Griggs et al. (1) proposed a specific set of criteria to diagnose FAPD in suspected cases of cicatricial alopecia. It requires a combination of clinical, trichoscopic, and histologic data (1). We believe this approach fully applies to our population and facilitates the appraisal of differential diagnosis (Figure 2) (1). According to those criteria, all subjects in our sample presented a symmetrical pattern of hair loss in androgen-dependent areas of the scalp (1). Our trichoscopic findings were perifollicular scale and follicular keratosis, loss of follicular openings, hair diameter variability, single hairs per follicular unit, and perifollicular erythema, which correspond to all the listed trichoscopic criteria (Figure 3) (1). Finally, all histologic criteria, virtually the same as those used for AGA and LPP, were fulfilled in our study from one or two 4 mm-punch biopsies (1). The vertical/horizontal sections revealed concentric perifollicular lamellar fibrosis, lymphohistiocytic infiltrate (predominantly in the upper region of the hair follicle), fibrotic follicular tracts, follicular miniaturization, and interface dermatitis (1–3). In the present study, concentric perifollicular lamellar fibrosis was considered an absolute diagnostic criterion for inclusion. Despite the heterogeneity in biopsy orientation, diagnostic confidence was maintained because this hallmark histopathological feature was identified in 100% of cases, allowing consistent clinicopathological correlation across all participating centers. The optimal treatment for FAPD is yet to be determined. The information available is based on case reports and retrospective studies, with unclear conclusions about follow-up and prognosis (1, 2, 4). The treatment aims to halt inflammation, prevent disease progression and scarring alopecia, and reverse the miniaturization of the hair follicle, an apparent trigger in genetically predisposed subjects (1, 4). In our study, we observed the use of several therapies including topical, intralesional, and systemic anti-inflammatory agents, growth-stimulating agents, and anti-androgenic agents, all of which are involved in the pathophysiology of this entity.

Table 1 shows a clinical, trichoscopic and histological comparison as well as demographic values from our study and from some of the case series reviewed in this article.

**Table 1:** Comparison of demographics and clinical characteristics for our study sample; comparisons of our study sample with findings from Zinkernagel and Trüeb (3) and Mardones et al. (5).

Variables	Statistics/categories	Statistical values		
		Our study sample	Zinkernagel and Trüeb (3)	Mardones et al. (5)
Demographic				
Sex, *n* (%)	Male	46 (100)	4 (21.1)	4 (30.8)
	Female	–	15 (78.9)	9 (69.23)
Mean age	Male	39	54	37
	Female	–	60	47.8
Clinical manifestation				
Pattern of scalp hair loss	FPHL, *n* (%)	16 (34.8)	13 (68.4)	–
	MPHL, *n* (%)	30 (65.2)	6 (31.57)	–
Scalp dysesthesia, *n* (%)		2 (6)	37%	12 (84.6)
Trichoscopic findings				
	Perifollicular scaling, *n* (%)	44 (96)	–	13 (100)
	Loss of follicular openings, *n* (%)	41 (89)	–	5 (38.5)
	Variability of hair shaft diameter, *n* (%)	41 (89)	–	–
	Single hair follicles, *n* (%)	38 (83)	–	–
	Perifollicular erythema, *n* (%)	32 (70)	–	13 (100)
Histopathological findings				
	Concentric perifollicular lamellar fibrosis, *n* (%)	46 (100)	13 (68.4)	–
	Lymphohistiocytic infiltrate, *n* (%)	45 (98)	14 (73.7)	–
	Follicular fibrosis tracts, *n* (%)	29 (66)	–	–
	Follicular miniaturization, *n* (%)	29 (54)	10 (71)	

FPHL, Female Pattern Hair Loss; MPHL, Male Pattern Hair Loss.

The limitations of this study are related to its retrospective design. The sample processing techniques for histopathological evaluation were not fully standardized across centers: only 24% of patients had both vertical and horizontal sections available, while the remaining cases were assessed using a single biopsy orientation, predominantly vertical sections. To our knowledge, this represents the largest case series on male FAPD to date, and the largest composed exclusively of Latin American patients.

## 5 Conclusion

In summary, fibrosing alopecia in a pattern distribution (FAPD) remains a condition with limited epidemiological characterization. Our findings suggest a trend toward an earlier age of onset among Latin American men with FAPD, showing similarities to reports in Asian populations rather than those from European and North American cohorts. However, this observation should be interpreted with caution, as it may be influenced by differences in referral patterns, diagnostic awareness, and access to health care. We acknowledge the challenge of diagnosing male patients with FAPD, as they often clinically resemble AGA in their classic loss pattern. The diagnostic criteria proposed by Griggs et al. (1) proved valuable in assessing the clinical, trichoscopic, and histological presentation of the patients in this study.

To our knowledge, this study represents the largest case series of male FAPD documented thus far. Our exploration of the epidemiological details of this challenging condition aims to contribute to a better understanding of FAPD and potentially improve patients’ quality of life.

## Data Availability

The original contributions presented in the study are included in the article/supplementary material, further inquiries can be directed to the corresponding author.
